# Impact of resilience enhancement on communication apprehension among health communication sciences students during the COVID-19 pandemic: an experimental study

**DOI:** 10.1186/s12909-025-07975-1

**Published:** 2025-10-02

**Authors:** Nisreen Naser Al Awaji, Eman M. Mortada, Rania Alkahtani

**Affiliations:** 1 Department of Health Communication Sciences, College of Health and Rehabilitation Sciences, Princess Nourah bint Abdulrahman University, P.O Box 84428, Riyadh, 11671 Saudi Arabia; 2 Family and Community Medicine department, College of Medicine, Princess Nourah bint Abdulrahman University, P.O Box 84428, Riyadh, 11671 Saudi Arabia

**Keywords:** Communication apprehension, Resilience, Health communication sciences, COVID-19, Speech-language pathology, Audiology, Higher education, Psychological intervention

## Abstract

**Background:**

Communication apprehension (CA) poses a significant barrier for students transitioning to university life, particularly in health communication fields such as speech-language pathology and audiology. The COVID-19 pandemic exacerbated communication challenges through prolonged remote learning. Psychological resilience has been identified as a protective factor that may help mitigate these difficulties.

**Methods:**

This study aimed to develop and assess a resilience-focused intervention designed to improve communication apprehension among undergraduate students in the Department of Health Communication Sciences (HCS) and to examine the relationship between resilience and CA. A quasi-experimental pre-post design was implemented. Eighty-five female students completed validated scales measuring psychological resilience (Connor-Davidson Resilience Scale; CD-RISC-10) and communication apprehension (Personal Report of Communication Apprehension; PRCA-24) before and after participating in a resilience-enhancing intervention. Paired t-tests were conducted to assess changes, and significance was set at *p* < .05.

**Results:**

Significant improvements were observed in key resilience domains, including flexibility (*p* < .001), self-efficacy (*p* < .001), and cognitive focus (*p* = .003), along with a significant increase in overall resilience scores (*p* = .003). CA significantly decreased in the group discussion, meetings, and interpersonal conversation contexts (*p* < .001 for each). However, no significant reduction was observed in public speaking apprehension (*p* = .644). Post-intervention, total CA showed significant positive correlations with meeting (*r* = .270, *p* < .05), interpersonal conversation scores (*r* = .240, *p* < .05), and public speaking scores (*r* = .414, *p* < .01). Conversely, total PRCA-24 was negatively correlated with both group discussion (*r* = − .357, *p* < .01) and resilience scores (*r* = − .450, *p* < .01). Regression analysis revealed that improvement in resilience significantly predicted a reduction in CA scores (*p* = .036).

**Conclusion:**

Resilience-enhancement interventions may be effective in reducing CA among students in the department of HCS, particularly in interactive contexts. Given the critical role of communication skills in healthcare professions, integrating resilience-building programs into undergraduate education could support both psychological well-being and professional development. Further research is recommended to specifically target public speaking anxiety and to validate these findings across diverse student populations.

## Introduction

The transition from secondary school to university represents a critical developmental stage for undergraduate students and remains a major concern for higher education institutions [1]. This period often presents multiple challenges as students adjust to new academic expectations, social environments, and increased personal responsibilities. Among the most prominent difficulties faced during this transition is the challenge of establishing interpersonal connections and maintaining effective communication within the university setting [2].

The COVID-19 pandemic introduced unprecedented disruptions to educational systems worldwide, further complicating students’ transition into higher education. In response to public health concerns, many universities rapidly shifted from traditional face-to-face instruction to online learning modalities [3]. While necessary, this transition limited opportunities for spontaneous and informal communication and in-person socialization, both of which are critical factors for healthy academic adjustment and interpersonal development [4, 5]. Even after pandemic restrictions eased, many institutions continued to offer hybrid or fully virtual courses, indicating that the communicative challenges introduced during the pandemic persisted beyond the immediate crisis [3, 6]. Consequently, students, particularly those entering university for the first time, experienced declines in social–emotional skills, such as emotional expressivity and control, compared to pre-pandemic cohorts [7]. This may have contributed to increased communication apprehension (CA), especially in online or hybrid classrooms, where students reported heightened anxiety around presentations and webcam use [8].

CA, defined as the fear or anxiety associated with real or anticipated communication with others [9], can significantly impair students’ academic performance, social integration, and future professional effectiveness. This challenge is particularly acute among students in health-related fields such as speech-language pathology and audiology, where communication is not merely a skill but a central component of professional practice [10]. Students in these programs are expected to engage in complex interpersonal exchanges, including counseling and client education, often in emotionally sensitive contexts [10]. A recent study at the Federal University of Sergipe, Brazil, which surveyed 1,332 health sciences students, found alarmingly high levels of CA, with female students and pharmacy students exhibiting the highest scores [11]. These findings align with broader observations in the literature, indicating that female students and students in programs heavily reliant on communication skills may be particularly vulnerable to communication challenges during times of crisis [12].

Several studies have explored the impact of remote learning on communication competencies. Dodd et al. [13] reported that students engaged in online courses struggled to communicate effectively with instructors and peers, diminishing their academic experience. Similar findings were echoed by Messman and Jones-Corley [14] and Bolatov et al. [15], who highlighted a link between high levels of CA and weakened interpersonal relationships in online learning contexts. The absence of natural, in-person communication opportunities appeared to magnify existing apprehension and anxiety among students.

Within this context, Al Awaji et al. [12] conducted a study specifically focusing on CA among female college students enrolled in the College of Health and Rehabilitation Sciences. The study population included students from the Department of Health Communication Sciences (HCS), which encompasses both speech-language pathology and audiology programs. Their findings revealed that 28.2% of the students experienced high levels of CA, with students from the HCS department showing the highest scores. This result was particularly noteworthy and concerning because students in this department are expected to develop robust communication skills essential for diagnosing and managing patients with communication and hearing disorders [16, 17]. These findings underscore the urgent need to address communication challenges among health communication students, who will eventually serve as healthcare providers where communication competence is critical for clinical effectiveness and patient outcomes.

In light of these challenges, increasing attention has been directed toward the role of psychological resilience in supporting students’ adjustment to university life and mitigating the adverse effects of CA [18, 19]. Resilience is defined as “The process and outcome of successfully adapting to difficult or challenging life experiences, especially through mental, emotional, and behavioral flexibility and adjustment to external and internal demands” [20]. It reflects the capacity to bounce back from adversity and maintain psychological well-being under stress.

Previous studies have indicated that resilience plays a pivotal role in students’ psychological adjustment and academic persistence [21–24]. Resilient students are more likely to demonstrate positive coping strategies, maintain higher academic self-concept, and exhibit greater psychological well-being. Importantly, resilience has also been associated with better interpersonal communication and lower anxiety in challenging contexts [25]. Students with higher levels of resilience are thus hypothesized to adapt more successfully to new learning environments, manage interpersonal challenges more effectively, and exhibit lower levels of CA, a relationship supported by findings that resilience significantly predicts reduced communicative anxiety in educational settings [18]. Despite the theoretical basis for linking resilience and communication outcomes, empirical research directly examining this relationship remains limited, particularly within the context of health sciences education. Few studies have systematically investigated whether resilience serves as a protective factor against CA among students navigating both the typical challenges of university transition and the extraordinary disruptions caused by the COVID-19 pandemic. Understanding this relationship could offer critical insights for the design of targeted interventions to enhance students’ communication skills, reduce anxiety, and support academic success.

Accordingly, the present study builds on earlier research conducted by Al Awaji et al. [12], which identified high levels of CA among students in the Department of HCS. However, unlike the previous cross-sectional work, this study advances the field by implementing and evaluating a structured, resilience-focused intervention designed to reduce CA and improve psychological resilience in this vulnerable student population.

Therefore, the current study aimed to:

1. Develop and implement an intervention to reduce CA among HCS students.
2. Evaluate its effectiveness in improving psychological resilience and communication outcomes, and.
3. Examine the potential relationship between resilience and CA.

To achieve these goals, the following research questions were addressed:

- To what extent is psychological resilience improved following participation in a resilience-focused intervention among undergraduate HCS students?
- How does participation in the intervention impact CA across interpersonal, group, meeting, and public speaking contexts?
- What is the nature of the relationship between changes in resilience and reductions in CA?

## Methods

### Study design

This study employed a quantitative, quasi-experimental, one-group pre-test/post-test design to assess the effects of a resilience-focused intervention on CA among undergraduate students. The research was conducted during the COVID-19 pandemic recovery period at the Department of HCS.

### Participants

A total of 85 undergraduate female students were recruited from the speech-language pathology and audiology programs at the Department of HCS. Participants were eligible if they were currently enrolled in the program and had participated in hybrid or online education formats during the pandemic. Demographic information collected included age, academic program, level/semester of study (6th, 9th, or 12th level), and cumulative GPA. A convenience sampling method was used.

### Ethical considerations

Ethical approval of the study was granted by the Institutional Review Board (IRB) at Princess Nourah bint Abdulrahman University (Approval No. 22–0665).

#### Informed consent

Informed consent was obtained electronically before the pre-intervention survey via Google Forms. The consent form clearly outlined the study purpose, procedures, and participants’ rights, including the option to withdraw at any time without penalty.

#### Voluntary participation

Participation in this study was entirely voluntary. Students were explicitly informed that their decision to participate or decline would have no impact on their grades, academic standing, or relationship with instructors.

#### Recruitment process

Recruitment and consent procedures were conducted by a research assistant unaffiliated with course instruction or grading responsibilities to mitigate potential concerns of coercion, particularly since participants were recruited from the researchers’ home department.

#### Confidentiality and data protection

All responses were anonymized using participant-generated codes to protect student identities and ensure confidentiality. Data were stored securely in password-protected files and were accessible only to the research team.

### Research instruments

#### Sociodemographic questionnaire

Students completed a structured sociodemographic questionnaire developed by the research team to capture relevant background information. The questionnaire included items on age, academic program (speech-language pathology or audiology), academic level (6th, 9th, or 12th semester), self-reported cumulative GPA, marital status, and any history of physical or mental health diagnoses. It also assessed participants’ exposure to online or hybrid learning during the COVID-19 pandemic. These data were collected to describe the sample and to identify baseline characteristics that could potentially influence levels of psychological resilience or CA.

The health-related section of the questionnaire included yes/no checkboxes to indicate any diagnosed physical or mental health conditions, with an optional open-text field for elaboration. In addition, students rated their perceived stress levels and coping abilities using 5-point Likert scales, ranging from 1 (not at all) to 5 (extremely). While these contextual variables were not included in the statistical analyses, they were reviewed descriptively to provide additional context for interpreting the relationship between resilience and CA.

#### Connor-Davidson resilience scale

Psychological resilience was assessed using the Connor-Davidson Resilience Scale (CD-RISC- 10), a validated 10-item questionnaire [26]. The Arabic-translated version was employed in this study to ensure complete comprehension [27]. Each item is rated on a five-point Likert scale, ranging from 0 (“Not true at all”) to 4 (“True nearly all of the time”), resulting in a total possible score between 0 and 40. Higher scores reflect greater resilience, while lower scores indicate lower resilience levels [28]. The scale evaluates critical components of resilience, such as personal flexibility, self-efficacy, emotional regulation, optimism, and cognitive focus during stressful situations [28]. In this study, the CD-RISC 10 demonstrated good internal consistency, with a Cronbach’s alpha coefficient of 0.85.

#### Personal report of CA

CA was measured using the Personal Report of Communication Apprehension (PRCA-24), a well-established instrument developed by McCroskey [9] and McCroskey et al. [29]. To ensure full comprehension, the Arabic-translated version was employed in this study and demonstrated high reliability, with a Cronbach’s alpha of 0.87 [30]. The PRCA-24 assesses CA across four domains: group discussion, meetings, interpersonal conversations, and public speaking. Each item is rated on a five-point Likert scale, with higher scores indicating greater CA. Subscale scores range from 6 to 30, and total scores range from 24 to 120.

When interpreting the pre- and post-intervention results, it is important to remember the inverse scoring direction: higher scores on the CD-RISC-10 reflect greater psychological resilience, while higher scores on the PRCA-24 indicate greater CA.

### Intervention

A structured, semester-long, resilience-focused intervention was designed and implemented to enhance psychological resilience and reduce CA among undergraduate students. The intervention was developed de novo by a multidisciplinary faculty team to address the specific needs of students in the HCS programs during the COVID-19 pandemic recovery phase. While the structure and content were informed by evidence-based principles from the resilience literature, particularly the conceptual framework of the CD-RISC 10 [26–28], the sessions were not directly adapted from a single pre-existing manualized program. Instead, the faculty drew upon relevant published interventions targeting university students’ resilience, communication challenges, and mental well-being, in addition to their own clinical and teaching expertise. Content validity was enhanced through iterative reviews and consultation with subject matter experts in communication sciences and clinical psychology to ensure psychological accuracy, developmental appropriateness, and alignment with the target domains of resilience.

The intervention was delivered in person to small student groups (10–15 students per group), once a week over a period of 10 weeks, with each session lasting approximately 60 min. Each session followed a consistent structure: a short introductory presentation, guided discussion, applied activity or scenario-based exercise, and a brief reflection. The instructional strategy primarily relied on PowerPoint-based lectures, case examples, and interactive discussions. Content delivery was standardized across all groups using a shared slide deck and facilitator guide.

The intervention content addressed the five core domains of resilience as measured by the CD-RISC 10:

1. Flexibility: adaptation to change, reframing negative experiences, based on evidence linking cognitive flexibility to resilience and self-regulation [31];
2. Self-efficacy: confidence-building, goal-setting, and managing academic and communication challenges, supported by literature linking self-efficacy to academic motivation and resilience [32];
3. Emotion regulation: managing anxiety, identifying emotional triggers, and basic cognitive behavioral therapy (CBT) techniques, informed by systematic reviews of university mental health interventions [33];
4. Optimism: use of positive psychology tools such as the Best Possible Self writing exercise, gratitude journaling, and strengths-based reflection, with documented effects on subjective well-being and resilience [34–36];
5. Cognitive focus: attention control and stress-coping strategies using mindfulness and present-focused techniques [33].

All instructional content was collaboratively developed, reviewed by faculty experts. Although the complete intervention was not pilot-tested as a unit, several components had been previously implemented in co-curricular workshops, and feedback from those sessions informed refinements to the session flow, delivery style, and activity structure.

All five domains received equal instructional time and attention. To address CA, specific sessions incorporated strategies relevant to interpersonal communication (e.g., listening, initiating conversations), group discussion dynamics, and participation in meetings. Although public speaking was conceptually included, students did not engage in live speaking practice or deliver real-time presentations, which may have limited the impact of the intervention on public speaking anxiety.

Sessions were facilitated by trained faculty members from the in speech-language pathology and audiology programs. While these facilitators were not licensed mental health professionals, they had relevant experience in clinical teaching and student support. To ensure ethical alignment and content accuracy, a professor in clinical psychology reviewed the full set of intervention materials, advised on facilitation strategies, and confirmed that the program remained within appropriate non-clinical boundaries. Facilitators also attended a one-hour orientation session conducted by a senior faculty member in communication sciences, which included a walkthrough of the curriculum, a discussion of instructional objectives, and training on standardized delivery methods. These measures were implemented to ensure fidelity, ethical scope, and consistency across sessions while maintaining the educational focus of the intervention.

### Data collection procedures

Data collection occurred in two phases: pre-intervention and post-intervention. All assessments were administered online using Google Forms to ensure accessibility and reduce logistical burden.

- Pre-Intervention Phase: Students completed the sociodemographic questionnaire, the CD-RISC 10, and the PRCA-24 one week before the intervention began. Each participant was assigned a unique anonymized code to link pre- and post-intervention responses while preserving confidentiality. Surveys were completed independently by students and submitted electronically.
- Post-Intervention Phase: Within one week following the final intervention session, students were asked to complete the same set of instruments (CD-RISC 10 and PRCA-24) using their previously assigned anonymized codes. Data were submitted remotely, and participants were instructed not to discuss their responses with peers in order to reduce response bias.

Both data collection phases were supervised remotely by the research team to ensure procedural consistency and integrity. Survey links were password-protected, and only members of the research team had access to the data. Data were stored securely in accordance with the ethical protocols approved by the Institutional Review Board.

### Statistical Analysis

Data analysis was performed using the Statistical Package for the Social Sciences (SPSS), version 20 (IBM Corp., Chicago, IL, USA). Descriptive statistics (mean, standard deviation, frequency, percentage) were used to summarize demographic characteristics.

- The normality of data distribution was assessed using the Kolmogorov–Smirnov test.
- Internal consistency reliability of the CD-RISC 10 and PRCA-24 was confirmed by calculating Cronbach’s alpha coefficients, with α ≥ 0.70 considered acceptable.
- Paired sample t-tests were used to compare pre- and post-intervention scores of resilience and CA.
- Pearson’s correlation coefficients were computed to examine the relationships between post-intervention total CA scores and resilience, as well as CA subdomains (group discussion, meetings, interpersonal conversations, and public speaking).
- Multiple linear regression analysis was then conducted to identify post-intervention variables that significantly predicted total CA scores. Statistical assumptions for regression analysis (linearity, homoscedasticity, independence of residuals, and multicollinearity) were checked and met prior to analysis.
- Statistical significance was set at *p* <.05.
- Changes in scores were illustrated using boxplots to visualize distribution and medians across pre- and post-intervention phases.

## Results

### Participant characteristics

**Fig. 1:**
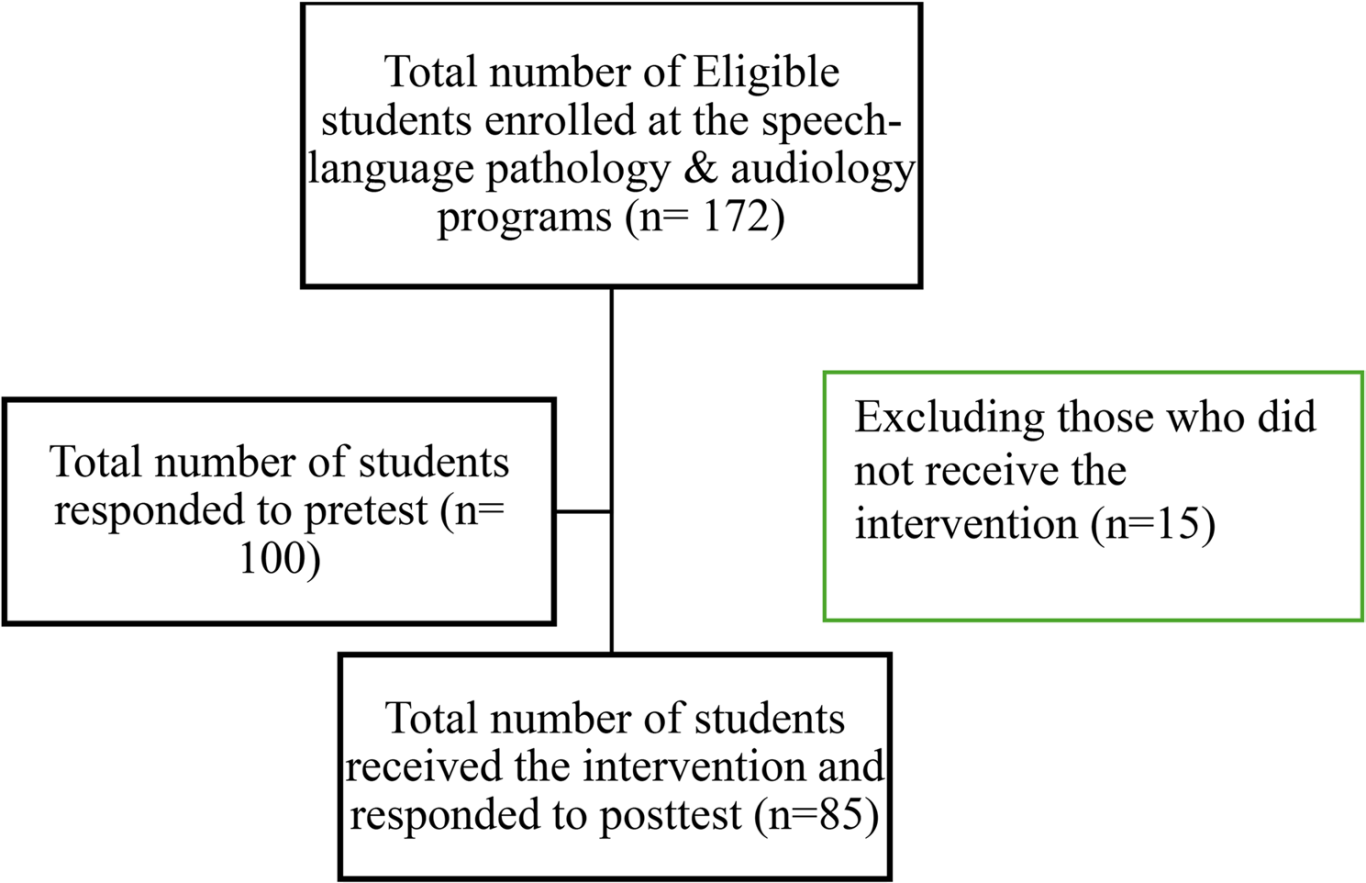
Flowchart illustrating the number of participants who were recruited, responded, and completed the intervention

A total of 85 female students participated in the study. All participants completed both pre- and post-intervention assessments, and no missing data were observed. The flow of participants through the study process, from recruitment to post-intervention assessment, is summarized in Fig. 1. The participants were distributed across two programs: speech-language pathology (52.9%) and audiology (47.1%). The majority were at the 6th academic level (57.6%), followed by the 9th (29.4%) and 12th (12.9%) levels. Regarding academic performance, 64.7% had a cumulative GPA of 4.3 or higher. Table 1 summarizes the personal characteristics of the study participants. Both the CD-RISC 10 and the PRCA-24 demonstrated good internal consistency in this sample, with Cronbach’s alpha coefficients of 0.85 and 0.87, respectively.

**Table 1:** Personal characteristics of the study participants (*n* = 85)

Characteristics/Responses	No	%
Program		
o Speech-language pathology	45	52.9
o Audiology	40	47.1
level		
o 6th	49	57.6
o 9th	25	29.4
o 12th	11	12.9
GPA		
o < 4.3	30	35.3
o ≥ 4.3	55	64.7
Total	85	100.0

### Resilience outcomes

The intervention led to significant improvements in several domains of resilience, as assessed by the CD-RISC 10.

As shown in Table 2:

**Table 2:** Comparison of mean scores for the five resilience domains, as measured by the CD-RISC-10 subscales, pre- and post- the resilience-focused intervention (*n* = 85). Higher scores indicate greater resilience in each domain

	M ± SD		Paired test results		
	Pre-intervention	Post- intervention	Paired Differences M ± SD	T	*P* value
Flexibility	4.85 **±** 1.21	5.78 **±** 1.80	− 0.93 ± 2.15	−3.989	< 0.001*
Sense of self-efficacy	7.67 ± 2.47	8.51 **±** 1.98	− 0.84 ± 2.30	−3.343	< 0.001*
Ability to regulate emotion	2.67 ± 1.15	2.84 ± 0.99	− 0.17 ± 1.50	−1.010	0.315
Optimism	8.33 ± 2.01	8.82 ± 2.21	− 0.49 ± 2.79	−1.634	0.106
Cognitive focus	2.90 ± 0.96	3.34 ± 0.87	− 0.44 ± 0.1.29	−3.099	0.003*
Overall CD-RISC 10 score	26.86 ± 5.96	28.85 ± 4.97	−1.99 ± 6.03	−3.042	0.003*

*M* mean, *SD*: standard deviation,* CD-RISC,* Connor-Davidson Resilience Scale

* p value of paired t,

- Flexibility significantly improved (mean increase from 4.85 ± 1.21 to 5.78 ± 1.80; *p* <.001; Cohen’s d = 0.43),
- Sense of self-efficacy also improved significantly (mean increase from 7.67 ± 2.47 to 8.51 ± 1.98; *p* <.001; Cohen’s d = 0.36),
- Cognitive focus under stress showed a significant increase (mean increase from 2.90 ± 0.96 to 3.34 ± 0.87; *p* =.003; Cohen’s d = 0.34).

No statistically significant changes were observed in the Ability to regulate emotion (*p* =.315) and Optimism (*p* =.106).

**Fig. 2:**
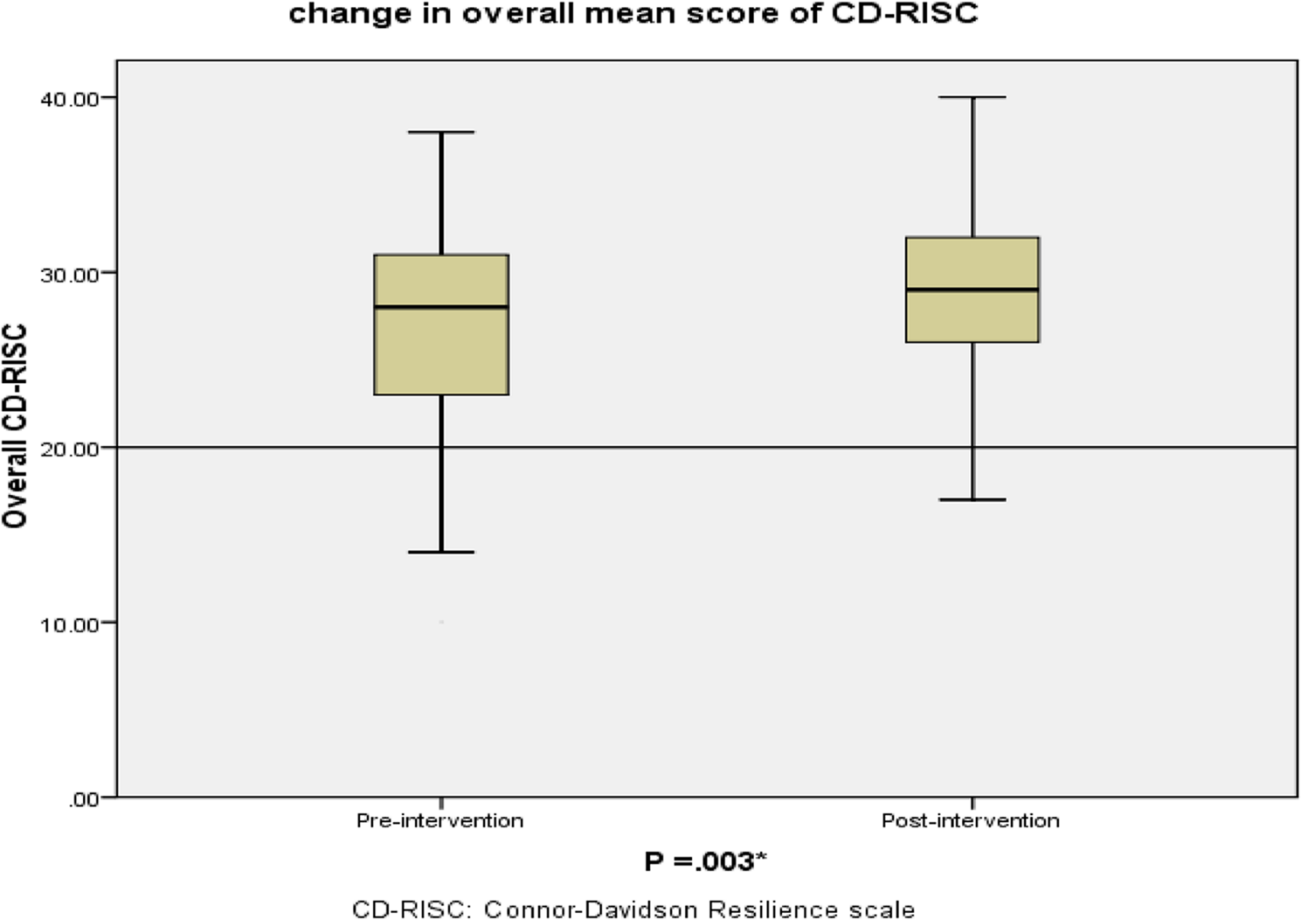
Boxplot illustrating the distribution of total resilience scores pre- and post-intervention, as measured by the CD-RISC-10

Overall, the mean score on the CD-RISC 10 significantly increased from 26.86 ± 5.96 pre-intervention to 28.85 ± 4.97 post-intervention (*p* =.003; Cohen’s d = 0.33), indicating a small to moderate positive effect of the resilience-building intervention. Figure 2 illustrates the changes in overall resilience scores using a boxplot representation.

### CA outcomes

Significant improvements in CA were observed across several domains of the PRCA-24. Specifically, as shown in Table 3:

**Table 3:** Comparison of mean scores for the four CA domains pre- and post-intervention (*n* = 85), as measured by the PRCA-24. Lower scores indicate reduced CA

Domains of PRCA-24	M ± SD		Paired test results		
	Pre-intervention	Post-intervention	Paired Differences M ± SD	t	*P* value
Group discussion	16.19 ± 4.43	21.68 ± 5.78	−5.49 ± 8.25	−6.143	< 0.001*
Meetings	15.14 ± 3.85	18.57 ± 2.48	−3.44 ± 5.41	−5.854	< 0.001*
Interpersonal Conversations	16.49 ± 2.69	22.81 ± 4.19	−6.32 ± 4.39	−13.270	< 0.001*
Public Speaking	17.77 ± 4.23	18.02 ± 5.23	− 0.25 ± 4.92	− 0.463	0.644
Overall PRCA-24	65.60 ± 5.55	81.09 ± 14.76	−1.55 ± 16.61	−8.600	< 0.001*

*M* mean *SD* standard deviation, *PRCA-24* Personal Report Communication Anxiety

* p value of paired t,

- Group discussion scores increased significantly (the mean increased from 16.19 ± 4.43 to 21.68 ± 5.78; *p* <.001; Cohen’s d = 0.67),
- Meetings scores increased significantly (the mean increased from 15.14 ± 3.85 to 18.57 ± 2.48; *p* <.001; Cohen’s d = 0.64),
- Interpersonal conversations showed a large improvement (the mean increased from 16.49 ± 2.69 to 22.81 ± 4.19; *p* <.001; Cohen’s d = 1.45).

In contrast, public speaking scores did not show a statistically significant change (*p* =.644).

**Fig. 3:**
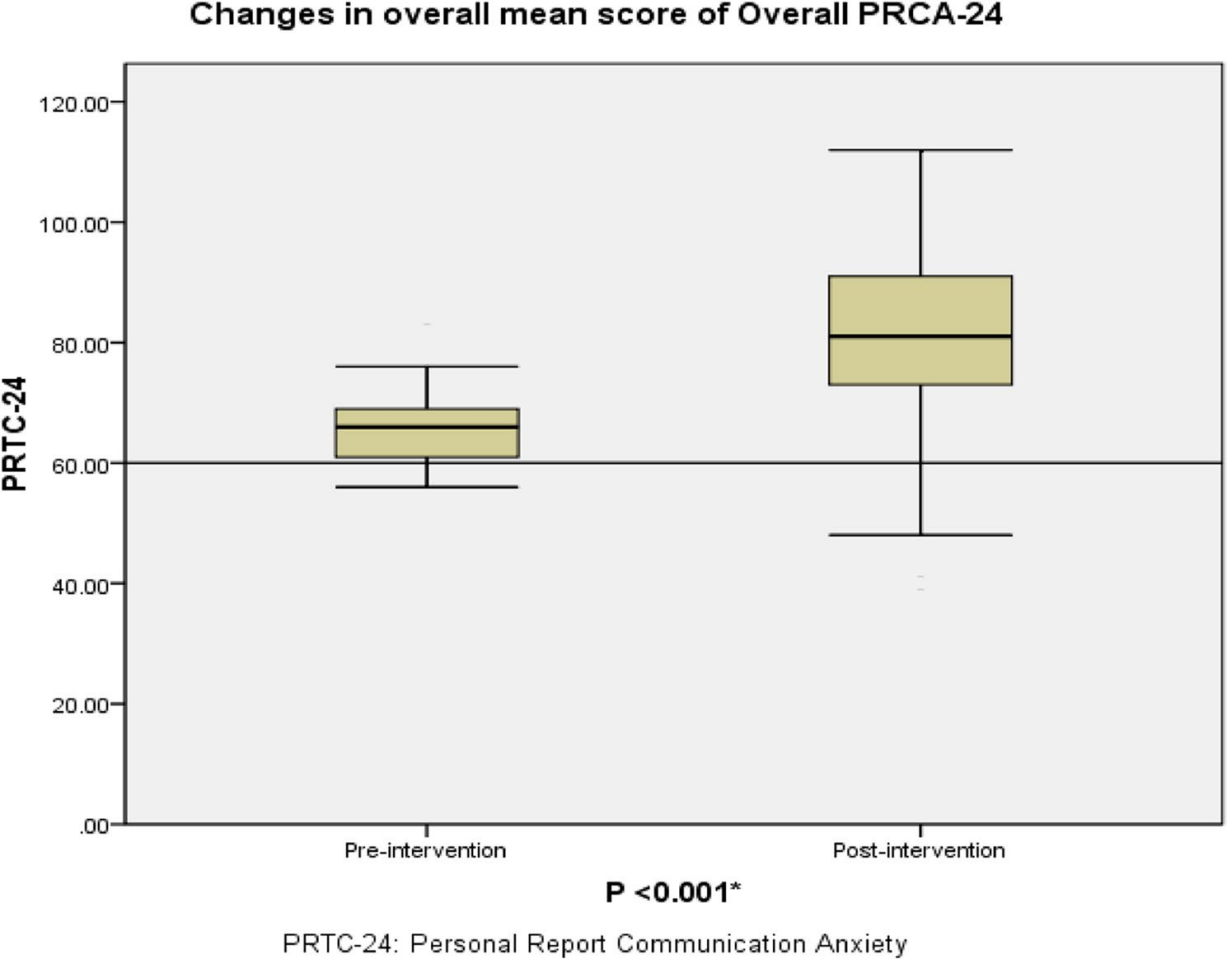
Boxplot illustrating the distribution of total CA scores pre- and post-intervention, as measured by the PRCA-24

The overall PRCA-24 total score significantly improved from 65.60 ± 5.55 to 81.09 ± 14.76 post-intervention (*p* <.001; Cohen’s d = 0.93), reflecting a large effect size for improvement in CA. The changes in overall CA scores are illustrated in Fig. 3.

Table 4 shows significant positive correlations between total CA and both post-intervention meeting and interpersonal conversation scores (*r* =.270 and 0.240, respectively; *p* <.05). Additionally, a significant positive correlation was observed between total PRAC-24 scores and post-intervention public speaking scores (*r* =.414, *p* <.01). Conversely, significant negative correlations were found between total PRAC-24 scores and both post-intervention group discussion and post-intervention resilience scores (*r* = −.357 and − 0.450, respectively; *p* <.01), indicating that improvements in group discussion and resilience were associated with reductions in CA.

**Table 4:** Correlation between mean scores of post-intervention total CA scale (PRAC-24) and selected variables

Variable	PRCA-24
Program	*r* =.135
Academic level	*r* =.003
GPA	*r* =.017
Post intervention Resilience	*r*=-.450**
Post intervention Group discussion	*r* = −.357**
Post intervention Meetings	*r* =.270*
Post intervention Interpersonal Conversations	*r* =.240*
Post intervention Public Speaking	*r* =.414**

* GPA* Graded Point Average, *PRCA-24,* Personal Report of Communication Anxiety, r Person correlation coefficient

*p <.05, **p <.01,

Regarding predictors of post-intervention CA, Table 5 shows that improvement in post-intervention resilience significantly predicted a reduction in CA scores (*p* =.036). No other predictors reached statistical significance.

**Table 5:** Regression analysis of variables predicting total post-intervention CA

	Unstandardized Coefficients		Standardized Coefficients	T	*P* value	95.0% C I interval for B	
Variable	B	SE	β				
						Lower bound	Upper bound
Constant	79.072	22.339		3.540	0.001	34.607	123.537
Post intervention Resilience	− 0.905	0.527	− 0.236	−1.72	0.040	−1.954	0.144
Post intervention Group discussion	− 0.404	0.446	− 0.121	− 0.91	0.368	−1.293	0.484
Post intervention Meetings	0.368	0.327	0.124	1.124	0.6	1.283	2.020
Post intervention Interpersonal Conversations	− 0.032	0.674	− 0.006	− 0.048	0.962	−1.373	1.308
Post intervention Public Speaking	0.685	0.424	0.196	1.615	0.110	− 0.159	1.530

B: unstandardized beta” regression coefficient”, β: standardized beta,* SE* Standard error, *C*I confidence interval

*P ≤ 0.05 is considered statistically significant

### Summary of findings

The resilience-focused intervention yielded statistically significant, small-to-moderate improvements in overall psychological resilience. It also produced large, statistically significant reductions in CA, particularly in group discussions, meetings, and interpersonal conversations. In contrast, public speaking anxiety did not improve significantly, suggesting that additional targeted strategies may be required for this domain. Post-intervention analyses demonstrated that higher resilience scores were significantly associated with lower total CA scores, while regression analysis identified post-intervention resilience as the only significant predictor of total CA, highlighting its central role in reducing communication apprehension.

## Discussion

The present study explored the extent to which a resilience-focused intervention could enhance psychological resilience and reduce CA among undergraduate students in the Department of HCS, as well as the relationship between changes in resilience and CA. Overall, the findings suggest that enhancing psychological resilience may be associated with lower levels of communication-related anxiety, particularly in interpersonal settings. This is especially relevant for students preparing for clinical roles in speech-language pathology and audiology, where effective communication is a core professional competency. Post-intervention analyses confirmed a significant negative relationship between resilience and total CA, and regression analysis identified resilience as the only significant predictor of total CA, underscoring its potential role as a key mechanism for reducing communication-related anxiety.

Statistically significant improvements were observed in three key resilience domains: flexibility, self-efficacy, and cognitive focus, following the intervention. These results are consistent with recent research identifying resilience as a buffer against academic stress and psychological strain [37]. Flexibility facilitates adaptation to unpredictable environments, such as those created by the COVID-19 pandemic. Self-efficacy, defined as the individual’s belief in their ability to execute tasks and manage challenges as conceptualized in Bandura’s theory [38], may have played a central mediating role, where improved confidence in managing communication demands led to greater reductions in CA than would be expected from resilience alone. Although improvements in emotion regulation and optimism did not reach statistical significance, positive trends were observed, particularly in optimism (Table 2). This finding suggests that these traits may require longer-duration interventions or more targeted therapeutic strategies, such as cognitive-behavioral training, an approach supported by evidence that resilience and self-efficacy mediate the impact of anxiety and depression on burnout [26, 39].

The intervention also led to significant reductions in CA in the domains of group discussion, meetings, and interpersonal conversations. These findings are particularly valuable in health communication-related programs, where interpersonal interaction is essential for patient-centered care. Prior research by de Araújo et al. [11, 13] has shown that reduced face-to-face interaction during the pandemic negatively impacted students’ communication confidence, reinforcing the need for structured, skill-building interventions. This aligns with our finding of a significant negative correlation between group discussion CA and total CA, indicating that gains in this interactive communication domain may have contributed meaningfully to overall reductions in CA.

However, the lack of improvement in public speaking CA suggests that this domain maybe qualitatively different. Public speaking anxiety, often rooted in fear of negative evaluation and social judgment, may be less responsive to general resilience-building [9, 40, 41]. Unlike general interpersonal communication, which may benefit more directly from improvements in resilience and self-efficacy, public speaking confidence often develops through consistent, structured practice over time [42]. This aligns with the idea that certain domains of CA are more immediately modifiable via behavioral exposure and performance feedback [42, 43]. In our view, this form of communication requires gradual exposure and repeated opportunities for performance, making it less likely to respond to short-term interventions alone. Embedding such practice into the academic experience may be essential for producing meaningful, lasting reductions in public speaking apprehension. The positive correlation observed between public speaking CA and total CA further suggests that when public speaking anxiety is elevated, it can disproportionately drive overall apprehension levels, even when other domains improve.

It is important to highlight that although public speaking was conceptually included in the intervention curriculum, the mode of delivery may not have provided sufficient opportunity for real-time practice or performance-based exposure. As a result, students may not have had adequate chances to confront or manage the unique stressors associated with speaking before an audience. This delivery gap may help explain the lack of statistically significant change in public speaking apprehension despite gains in other communication domains. This perspective aligns with Lazarus and Folkman’s [44], who suggest that unless students perceive a high degree of control and coping capacity in public performance settings, resilience gains may not translate into reduced anxiety. Targeted strategies such as systematic desensitization, public speaking workshops, and, more recently, virtual reality therapy combined with counseling, may be effective approaches for addressing public speaking anxiety among students [45, 46].

These results reinforce prior findings on the relationship between psychological resilience and communication competencies [22, 24]. Students with enhanced resilience tend to report greater emotional regulation, self-confidence, and adaptability, which are associated with fewer barriers to communication. The observed improvement in both resilience and CA scores highlights the potential of resilience-building programs in supporting students during high-pressure academic transitions and post-pandemic recovery. Beyond the scope of communication, resilience has been associated with broader academic and psychosocial outcomes, including enhanced mental health, persistence, and peer relationships [23]. Thus, embedding resilience into the university curriculum could be explored as a potential multidimensional strategy to improve student well-being and professional preparedness.

It is worth noting, however, that the degree of improvement in CA was notably larger than the observed changes in resilience scores. While this may indicate a possible association between improved resilience and reduced CA, it underscores the limitations of drawing causal conclusions given the single-group design. These improvements in CA, particularly in group and interpersonal contexts, may have been influenced by other unmeasured factors or the communicative nature of the intervention sessions themselves. It is possible that the structure of the sessions, emphasizing communication tasks, group interaction, and scenario-based discussions, directly influenced CA through increased communication self-efficacy or comfort, rather than through broader psychological resilience changes. Although resilience may contribute to reduced CA, the data do not support a direct one-to-one correspondence between the two. This nuance should be taken into account when interpreting the study’s implications. Although the findings support prior literature linking resilience to communication competencies [22, 24], they also suggest that resilience may not act alone. Other factors, such as trait anxiety, prior communication experiences, social support, self-perceived communication competence, increased social exposure during the post-COVID transition, students’ prior communication experiences, and the gradual normalization of academic routines, may mediate or moderate the observed outcomes.

## Limitations of the study

This study has several limitations that should be considered when interpreting the findings. First, the absence of a control group restricts the ability to draw causal conclusions regarding the effectiveness of the intervention. While significant improvements were observed in both reseilience and CA, these changes must be interpreted as associations rather than definitive outcomes of the intervention. Second, the intervention was delivered exclusively to female undergraduate students from a single institution, which limits the generalizability of the results to broader populations, particularly that prior research suggests that gender can influence both resilience levels and communication styles [47]. The sample also included a high proportion of academically high-achieving students, potentially biasing the outcomes toward participants with stronger baseline coping capacities. Additionally, the short-term nature of the assessment prevents evaluation of the sustainability of intervention effects over time. Future research should incorporate longitudinal follow-up to examine the durability of observed gains. Third, although the intervention content was reviewed by a clinical psychologist to ensure conceptual accuracy and adherence to non-clinical boundaries, the sessions were delivered by faculty members with speech-language pathology and audiology backgrounds rather than licensed mental health professionals. While the facilitators had experience in clinical education and student support, the lack of formal psychological training may have limited the depth of therapeutic expertise and reduced the generalizability of the program to settings requiring clinical oversight.

Furthermore, several potentially influential variables, such as baseline anxiety, personality traits (e.g., introversion or extroversion), prior communication experiences, and levels of social support, were not measured or controlled These factors may have moderated or mediated the observed outcomes and should be considered in future research. Lastly, the study did not include a longitudinal follow-up to assess the sustainability of intervention effects over time. As such, it remains unclear whether the improvements in resilience and CA observed immediately after the intervention would persist in the long term. Longitudinal designs with follow-up assessments are recommended to evaluate the durability and real-world impact of similar interventions.

## Conclusion

This study provides evidence that resilience-enhancing interventions may be associated with reductions in CA among undergraduate students in HCS programs, particularly in domains requiring interpersonal interaction. Improvements in resilience, especially in flexibility, self-efficacy, and cognitive focus, were observed alongside gains in students’ confidence and adaptability in communication tasks, aligning with psychological theories of coping and behaviour change.

Higher post-intervention resilience was significantly associated with lower total CA and was the only significant predictor of overall CA. The negative correlation between group discussion CA and total CA supports the contribution of interactive communication skills to overall CA reduction, while the positive correlation between public speaking CA and total CA underscores the disproportionate impact of performance-based anxiety on overall CA.

Given the centrality of communication skills in speech-language pathology and audiology, the integration of resilience training into undergraduate education may represent a promising area for further exploration as a proactive and preventive strategy to strengthen both psychological well-being and professional readiness. However, the absence of improvement in public speaking apprehension underscores the need for more specialized interventions tailored to performance-based anxiety. These findings highlight the complexity of CA and the necessity of multi-modal intervention strategies in educational settings. Overall, promoting resilience emerges as a potential strategy for supporting students’ psychological well-being and professional skill development, particularly in the aftermath of pandemic-related educational disruptions.

## Future directions

Future research should consider expanding the intervention scope by developing and evaluating targeted approaches that directly address public speaking apprehension. Techniques such as systematic desensitization, structured public speaking training, and virtual reality–based exposure could be implemented alongside general resilience-building programs to address performance-based anxiety more effectively.

Replication of this study across multiple academic institutions with larger and more diverse student populations is necessary to enhance the external validity and generalizability of findings. Additionally, randomized controlled trials or matched control group designs are recommended to better establish causality and rigorously assess the effectiveness of resilience-based interventions.

To enhance transparency and replicability, future studies should implement standardized intervention manuals and facilitator training logs to ensure fidelity and consistency across different contexts. Longitudinal follow-up assessments at multiple time points post-intervention will be essential to evaluate the sustainability and long-term impact of improvements in both resilience and CA.

Future studies should also explore additional variables, such as baseline anxiety, personality traits (e.g., introversion), prior communication experiences, and social support, to better understand the factors influencing resilience and CA outcomes. Complementing quantitative findings with qualitative methods—such as interviews, focus groups, or reflective journals—could provide deeper insights into students’ lived experiences, coping mechanisms, and perceived barriers to communication, confidence, and resilience-building.

Finally, pilot programs that embed resilience training and communication skills development into existing academic or clinical curricula should be explored. Such integration may offer a sustainable, scalable approach to enhancing psychological preparedness, communication confidence, and professional readiness among healthcare students.

### Clinical trial number

not applicable.

## Data Availability

The data supporting the findings of this study are available from the corresponding author upon reasonable request.

## Abbreviations

CA: Communication apprehension
CD-RISC: Connor-Davidson Resilience Scale
HCS: Health Communication Sciences
PRCA: Personal Report of Communication Apprehension
